# Temporal–Spatial Differences of Nitrogen Source–Sink in Sediments of Wetland–River Connected System and Response Mechanism of Microbial Community Function

**DOI:** 10.3390/microorganisms14061216

**Published:** 2026-05-27

**Authors:** Zejun Shi, Yu Pan, Haojie Chen, Xueying Wang, Wei Huang, Lixin Li

**Affiliations:** 1 School of Environment and Chemical Engineering, Heilongjiang University of Science and Technology, Harbin 150022, China; 2 State Environmental Protection Engineering Center for Pollution Treatment and Control in Textile Industry, College of Environmental Science and Engineering, Donghua University, Shanghai 201620, China

**Keywords:** wetland–river system, nitrogen source–sink, microbial community, seasonal succession, driving mechanism

## Abstract

The spatiotemporal succession of microbial community structure influences sediment nitrogen (N) release. To compare the N release and microbial response between a large-scale wetland and its connecting rivers, sediment samples were collected across three seasons (October 2024, March 2025, and July 2025) and analyzed using sorption isotherms and sequencing to elucidate source–sink dynamics and microbial mechanisms. The results showed that the maximum sorption capacity (Q_max_, 9.931 mg/g) exhibited significant seasonal variation (March > July > October) and a vertical decreasing pattern (surface > middle > bottom). The Q_max_ of wetland sediments (SS) was generally higher than that of river sediments (SH). The N source–sink analysis indicated that SS consistently served as a stable N sink, while SH primarily served as a N source. Among them, the internal N release pressure in the rivers was highest in July, and a relatively high diffusion flux was still maintained in October. Microbial diversity was significantly higher in the warm seasons (July and October) than in spring, and spatially, diversity was higher in SS than in SH. *Proteobacteria* were the dominant phylum, with a relative abundance ranging from 8.11% to 35.59%. *Gammaproteobacteria* was the dominant class, with a maximum relative abundance of 28.36%. *Anaerolineae* in SH were significantly enriched in summer and autumn. The driving factors shifted from the physical particle size (D50) in spring to the organic load and nutrients (total nitrogen or total phosphorus) in summer, and then to the synergistic effect of pH and physical structure in autumn. Functional prediction indicated that the microbial functions in river channels evolved from reserve-type heterotrophic metabolism to high-activity energy metabolism, with the highest predicted potential observed in July. In contrast, the wetland consistently maintained steady-state regulatory functions centered on signal transduction and membrane transport.

## 1. Introduction

Nitrogen (N) is a key driver of water eutrophication, regulates the nutrient supply and transformation processes in aquatic ecosystems, and determines the health, stability and ecological balance of aquatic environments [1,2]. The adsorption, desorption and release of N in sediments constitute a critical process governing nutrient dynamics across entire aquatic ecosystems [3]. Exogenous external inputs, such as agricultural runoff and domestic sewage, and endogenous sediment internal release can influence N accumulation in aquatic environments [4]. Relevant data indicate that endogenous N release can serve as a nitrogen source in the overlying water [5]. Internal N release from sediments not only replenishes water-column nutrient pools but can also trigger cascading ecological effects such as algal blooms, thereby exacerbating eutrophication [6]. Meanwhile, N adsorption and desorption in sediments directly define the N source–sink function of aquatic ecosystems, playing an irreplaceable role in buffering external nitrogen inputs and maintaining water quality stability [7]. Therefore, clarifying the behavior dynamics and migration patterns of N in sediments is of great significance for the effective prevention and control of water eutrophication and environmental management. Wetlands and their connected rivers form a typical aquatic continuum characterized by intense material exchange and ecological coupling, where sediments serve as an important interface for N retention, transformation and release [8,9]. Intensified seasonal hydrological fluctuations and habitat heterogeneity have led to substantial differences in the physicochemical properties and microbial community structures between wetland sediments and riverine sediments, resulting in significant spatiotemporal variations in nitrogen release potential and migration pathways, exerting profound impacts on N homeostasis [10,11].

The spatiotemporal succession of microbial communities is increasingly recognized as a key driver of N release [12]. The key functional nitrogen cycle microbiota, including *Proteobacteria*, *Bacteroidetes* and *Acidobacteria*, jointly affect the sediment–aquatic N cycle metabolic pathways [13]. Microbial community composition, diversity and metabolic functions are highly sensitive to seasonal changes in temperature (T), pH, particle size, organic matter (OM) and nutrient availability [14]. The seasonal succession of microbial communities is closely related to the mineralization, nitrification, denitrification and N release of sediments [15]. In addition, submerged macrophytes can influence sediment redox conditions, root exudate distribution, and microbial functional gene expression, further regulating N transformation and source–sink conversion at the sediment–water interface [16,17]. However, most current research focuses on individual wetlands or isolated river reaches, and insufficient understanding exists regarding the cross-seasonal differences in nitrogen source–sink dynamics and microbial response mechanisms across wetlands and their connected rivers. In particular, the seasonal shifts in environmental drivers of nitrogen retention and release, the functional succession of microbial communities, and the coupling regulation of submerged plants on functional genes and sediment N source–sink conversion remain to be systematically elucidated. Therefore, it is meaningful to analyze habitat-specific seasonal differences in endogenous N release using in situ adsorption experiments, and to clarify how combined nitrogen stress and habitat properties shape core nitrogen-cycling microbial communities.

Based on the above research and existing theoretical foundations, four testable and directional hypotheses were proposed for this study: (1) Wetland sediments (SS) and river sediments (SH) differ in their nitrogen adsorption capacity and source–sink functions, and exhibit seasonal variations. (2) Microbial community diversity, composition, and structure differ significantly between SS and SH, and the seasonal succession of microbial communities is significantly correlated with the sediment nitrogen release potential and adsorption capacity. (3) The environmental driving factors of nitrogen adsorption–desorption and microbial community succession vary with seasons. (4) Microbial functional potential succession is correlated with sediment nitrogen source–sink conversion dynamics. Therefore, sediment samples were collected from a large wetland and its connected rivers across three representative seasons (October 2024, March 2025 and July 2025). Adsorption isotherms were used to characterize the N adsorption capacity and source–sink transition patterns, while 16S rRNA bioinformatic analysis was used to characterize the microbial community diversity, composition, and functional potential, and the hypotheses were statistically verified using correlation analysis.

Building on the above innovations, this study aims to clarify seasonal and vertical variations in the N adsorption capacity of wetland and riverine sediments, to identify temporal–spatial differences in N source–sink functions and internal N release risks between wetlands and rivers, to reveal the seasonal succession characteristics of the dominant microbial taxa and community diversity, and to explore the seasonal environmental driving mechanisms and the coupling relationship between microbial functional succession and sediment N source–sink dynamics. The results can provide a scientific basis for nitrogen pollution control, eutrophication mitigation, and ecological management in wetland–river connected aquatic systems, and also support functional gene regulation research and targeted endogenous pollution control.

## 2. Materials and Methods

### 2.1. Sample Collection and Processing Methods

This study divided the sampling area into two types, rivers (H) and wetlands (S), with 15 sampling points selected (8 rivers (H1–H8) and 7 wetlands (S1–S7)) in October 2024, March 2025 and July 2025. The specific location information (Figure S1 and Table S1) is provided in the Supplementary Materials. Wetland sediments were labeled as SS, while river sediments were labeled as SH. Stratified sampling was conducted at each sampling point using a columnar sediment sampler, with surface (0–10 cm), middle (10–20 cm), and bottom (20–30 cm) sediment samples collected according to predefined stratigraphic layers. Portable multi-parameter water quality analyzers were used on-site to measure the pH and dissolved oxygen (DO) in sediments at each sampling point. Collected samples were promptly stored in polyethylene self-sealing bags and transported back to the laboratory under low temperatures (<0 °C).

### 2.2. Sample Analysis Method

The sample analysis method is referred to in the Supplementary Materials.

### 2.3. Method of Microbial Analysis

Sediment total genomic DNA was extracted using a soil DNA extraction kit (MagaBio, Shanghai, China) following the manufacturer’s instructions. The DNA quality and concentration were verified using a NanoDrop ND-2000 spectrophotometer (Thermo Fisher Scientific, Waltham, MA, USA) and agarose gel electrophoresis. Only high-quality DNA samples (OD260/280 = 1.8–2.0, total DNA > 50 ng) were used for downstream PCR amplification.

The V3–V4 hypervariable region of the bacterial 16S rRNA gene was amplified using the universal prokaryotic primers 338F (5′-ACTCCTACGGGAGGCAGCAG-3′) and 806R (5′-GGACTACHVGGGTWTCTAAT-3′) [18,19]. PCR was performed in a 20 μL reaction system containing 10 μL 2× Taq PCR Master Mix, 0.8 μL of each primer (5 μM), 1 μL template DNA (10 ng/μL), and 7.4 μL sterile ddH_2_O. The PCR thermal program was set as follows: initial denaturation at 95 °C for 3 min; 30 cycles of denaturation at 95 °C for 30 s, annealing at 56 °C for 30 s, and extension at 72 °C for 45 s; and a final extension at 72 °C for 10 min.

PCR products were purified using AMPure XP magnetic beads (Beckman Coulter, Brea, CA, USA). Sequencing libraries were constructed using a library preparation kit, and the library concentration was quantified using a Qubit 4.0 fluorometer (Thermo Fisher Scientific, Waltham, MA, USA). All 16S rRNA gene amplicon sequencing was performed on the Illumina NovaSeq X Plus platform (PE150) at Shanghai Majorbio Bio-pharm Biotechnology Co., Ltd. (Shanghai, China), following standard Illumina protocols [20,21].

Raw paired-end reads were quality-filtered and trimmed using fastp with the default parameters to remove low-quality reads, adapter sequences, and ambiguous bases (N > 10%). Clean reads were merged using FLASH (version 1.2.11) and clustered into operational taxonomic units (OTUs) at a 97% similarity threshold using USEARCH (version 11). Taxonomic annotation was assigned to representative OTU sequences using the RDP classifier (Bayesian algorithm) against the SILVA 16S rRNA database (v138) with a confidence threshold of 70% [22].

The alpha diversity indices (Chao1 and Shannon) and beta diversity were calculated using QIIME2 (version 2022.2). The LEfSe, PCA, RDA, and Mantel tests were performed to reveal community variation and driving factors. Microbial functional prediction was conducted using PICRUSt2 based on high-quality 16S rRNA amplicon data to generate KEGG and COG functional profiles, with a strict E-value < 1.0 × 10^−5^ for annotation [23,24,25,26]. All bioinformatic analyses were completed on the Majorbio Cloud Platform (https://cloud.majorbio.com/ (accessed on 2 November 2025)) [27].

All sequencing data analyzed in this study will be downloaded from NCBI’s Sequence Read Archive after the article is accepted, with samples from three seasons of rivers and wetlands.

## 3. Results

### 3.1. N Sorption Parameters of Sediments from Wetlands and Rivers

**Figure 1:**
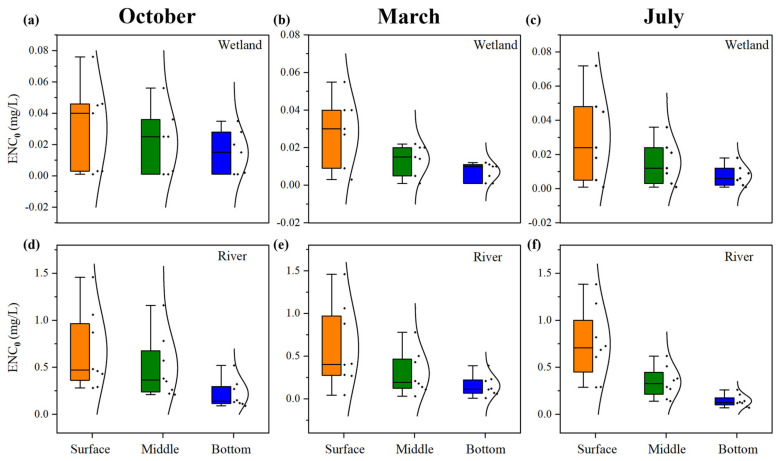
ENC_0_ of the wetlands in October (**a**), March (**b**), and July (**c**); and ENC_0_ of the rivers in October (**d**), March (**e**), and July (**f**).

In this study, the modified Langmuir model was used to calculate Q_max_ and ENC_0_, and the results are shown in Figure 1 and Figure S2. The Q_max_ values of SS in the three months were greater than those of the middle and bottom layers. Among them, the maximum Q_max_ of SS in March reached 9.931 mg/g. For the middle layer, Q_max_ values ranged from 0.236 to 8.211 mg/g across the three months, with an average sorption capacity of 1.559 mg/g in the wetland and 0.630 mg/g in the river, indicating a spatial pattern of wetland > river. For the bottom sediments, the highest mean Q_max_ (1.892 mg/g) was also observed in March, and the spatial pattern remained wetland > river.

From a temporal perspective, the average Q_max_ of the sediments in the study areas exhibited significant seasonal fluctuations, generally following the order: March (3.168 mg/g) > July (1.451 mg/g) > October (1.105 mg/g). In March, the Q_max_ values of the study area reached their peak, with an average surface sediment sorption capacity of 3.168 mg/g for SS, far exceeding those of the other two periods. Among all sites, S1 (the deep purification zone) exhibited the most outstanding performance, with a Q_max_ value of 9.931 mg/g from surface SS. Regarding the difference in sorption performance between summer and autumn, in July (summer), the Q_max_ values of surface SS decreased to 1.451 mg/g. Although high summer temperatures limited physical sorption to some extent, biological activity within the wetland was most vigorous during this season. Especially in the root-channel purification zones (S2, S3, and S7), the oxygen secretion by plant roots and rapid microbial turnover helped maintain a moderate sorption potential. In contrast, the average sorption capacity in October (autumn) was the lowest, with the Q_max_ values of surface SS averaging only 1.105 mg/g. In terms of vertical distribution, the sorption potential of all three periods strictly followed a decreasing pattern of surface > middle > bottom layer.

### 3.2. Differences of the N Source and Sink of the Sediments from Wetlands and Rivers

**Figure 2:**
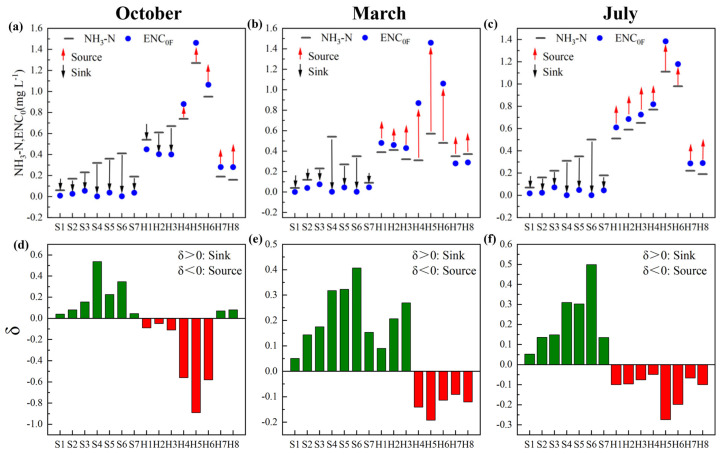
Differences in source–sink characteristics of the sampling points across different periods: NH_3_-N and ENC_0_ in October (**a**), March (**b**), and July (**c**); and δ in October (**d**), March (**e**), and July (**f**) (Green: sink; Red: source).

By comparing the dynamic relationship between ENC_0_ in sediments and the NH_3_-N concentration in overlying water, ENC_0_ represents the critical NH_3_-N concentration in the aqueous phase at which the sediment system reaches a dynamic equilibrium between adsorption and desorption; at this concentration, the net N flux between the sediment and water is zero, and the N source and sink characteristics of the study area during different periods was quantitatively distinguished using the ENC_0_ and δ method derived from isothermal adsorption experiments under laboratory conditions [28,29]. Figure 2a–c show that the N role of sediments in this region exhibits strong spatial heterogeneity and seasonal fluctuations. Spatially, there is a clear contrast between SS as a N sink and SH as a N source. The SS consistently acted as the N stable sink, and data showed that the NH_3_-N concentration of overlying water was generally higher than their corresponding ENC_0_ (δ > 0). In contrast, the ENC_0_ of SH was generally much higher than the NH_3_-N concentration of the overlying water (δ < 0).

From a temporal perspective, the intensity of the sediment as the N source varied significantly with the season. In March, the source role of the river areas was relatively weak. Some SHs (e.g., H1, H2, and H3) even exhibited positive δ values, temporarily shifting from source to sink. This may be related to the low microbial mineralization rate under low water temperatures in spring, resulting in relatively small amounts of adsorbed N accumulated in the sediments. July was the period with the highest internal N release pressure. The ENC_0_ values of SH generally increased, with point H5 maintaining a high ENC_0_ of 1.384 mg/L. In October, the source characteristics of the river areas remained significant, and points such as H4, H5, and H6 showed large negative δ values.

In addition, by comparing the N source and sink intensity (absolute δ value) across different seasons (Figure 2d–f), it was found that the N release characteristics of the study area sediments exhibited significant spatial heterogeneity and seasonal synchrony [30]. Spatially, SS (S1–S7) consistently acted as a stable dual sink, with all δ values positive and a relatively high N retention intensity (up to 0.537). In contrast, the SH (H1–H8) generally exhibited dual source characteristics, with point H5 being the core pollution source, where δ reached as low as −0.89 in October, reflecting a huge internal release pressure.

### 3.3. Microbial Community Diversity During N Release Process in Sediments

**Figure 3:**
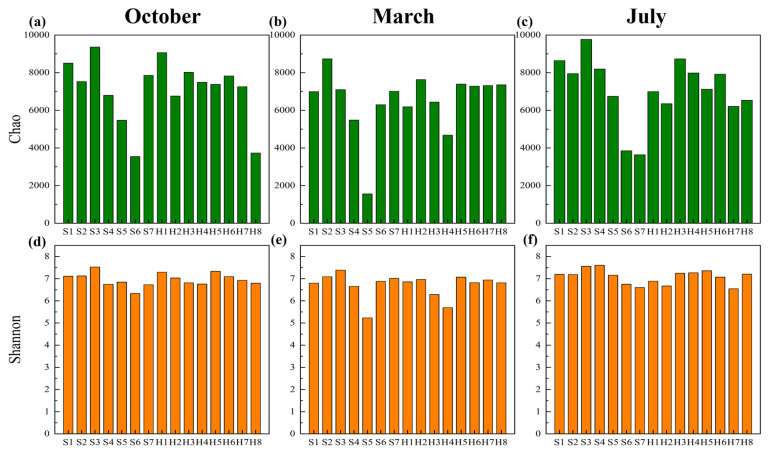
Microbial community diversity at the sampling sites across different periods: Chao in October (**a**), March (**b**), and July (**c**); and Shannon in October (**d**), March (**e**), and July (**f**).

In this study, the Chao index and Shannon index were used to characterize the species richness and diversity of the microbial communities in the sediments [31] (Figure 3). The results showed that the microbial community diversity in the study area displayed spatiotemporal variation. From a temporal perspective, the overall microbial diversity followed the trend: July 2025 ≈ October 2024 > March 2025. Microbial activity was high in July and October, with the Chao index at point S3 in October reaching 9365.83, the highest level of the year, and the Shannon index at various points in July generally ranging between 6.50 and 7.60. This indicates that warm seasons favor OM mineralization, and the abundant nutrient supply supports higher levels of microbial community richness. In March, due to low temperature constraints, community diversity dropped to its minimum. At some sampling points (e.g., S5), the Shannon index was only 5.23, and the Chao index also declined significantly, reflecting a simplification of community structure during the winter–spring transition.

Spatially, the microbial diversity followed the distribution pattern: interior of the wetland (S1–S3) > river (H1–H8). Regardless of the season, the Chao index at wetland interior points (especially S3) was significantly higher than that at the surrounding river. In contrast, the richness of SH was relatively low and more variable.

### 3.4. Microbial Community Composition at the Phylum and Class Level During the N Release Process of the Sediments

**Figure 4:**
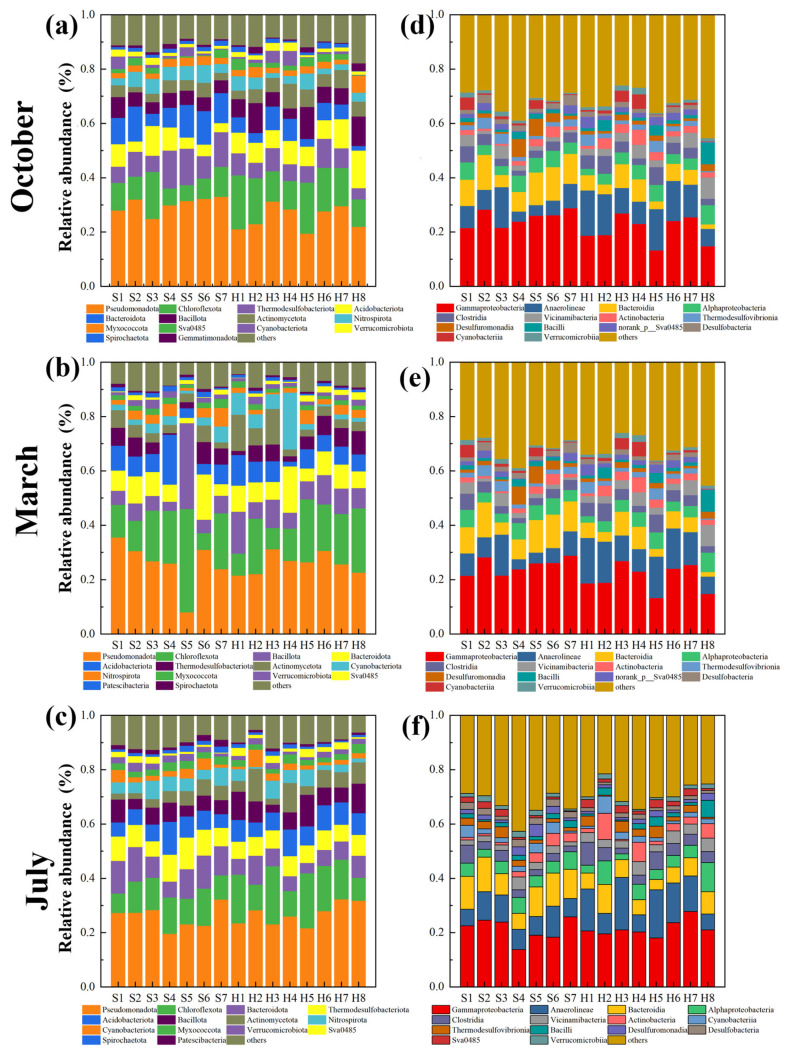
Relative abundances of microorganisms at individual sites across different periods: phylum level in October (**a**), March (**b**), and July (**c**); and class level in October (**d**), March (**e**), and July (**f**).

The microbial community composition of the sediments in the study area during the three periods is shown in Figure 4a–c. *Proteobacteria* was the dominant phylum across all sampling points, with relative abundances ranging from 8.11% to 35.59%. *Bacteroidota*, *Acidobacteriota*, and *Chloroflexota* also accounted for large proportions. Spatially, there were certain differences in the microbial community between SS and SH. In October, the abundance of *Proteobacteria* at S7 was the highest (33.02%), while the proportion of *Chloroflexota* at river point H5 (18.79%) was significantly higher than in other areas, indicating that the OM content and hydrodynamic conditions among different sites may strongly drive community evolution at the phylum level. In March, at point S5, the abundance of *Proteobacteria* (8.11%) was significantly lower than the regional average. This may be related to the local low water level in the wetland during the dry spring season and the low sediment temperature, conditions that limited the proliferation of some aerobic or facultative heterotrophic microorganisms [32]. In July, SH showed strong synchrony in the proportions of *Proteobacteria* and *Chloroflexota*.

At the class level (as shown in Figure 4d–f), *Gammaproteobacteria* was the absolutely dominant class across the study area, with a maximum relative abundance of 28.36%. In October, *Anaerolineae* ranked second in abundance after *Gammaproteobacteria*, showing obvious enrichment at river points H5 and H6 (15.21% and 14.89%, respectively). This distribution pattern is closely related to the physicochemical properties of the sediments. In March, the proportion of *Anaerolineae* increased significantly at points such as H5 and H6 (both exceeding 20%). In July, the abundance of *Cyanobacteriia* was significantly higher than in the previous two periods, reaching 4.44% at point S1. This change is closely related to the strong summer light, high water temperature, and abundant nutrient supply. At the same time, the sustained high proportion of *Anaerolineae* at river points (e.g., H5, 17.72%) indicates that the river sediments maintained a strong anaerobic OM degradation process in summer.

LEfSe analysis identified season-specific microbial biomarkers (LDA > 3, *p* < 0.05) (Figure S3) [33]. In July, *Cyanobacteriota* were significantly enriched, indicating enhanced photosynthetic activity under a high temperature and nutrient loading. March was characterized by the enrichment of *Thermodesulfobacteriota* and *Margulisbacteria*, reflecting an oligotrophic and anaerobic environment. October was distinguished by *Myxococcota* and *Deferrisomatota*.

### 3.5. RDA and PCA Analysis of Microbial Community

**Figure 5:**
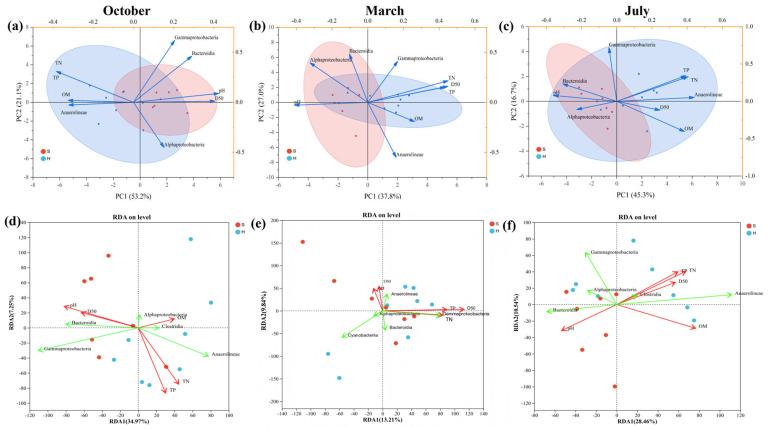
PCA ((**a**): October; (**b**): March; and (**c**): July) and RDA ((**d**): October; (**e**): March; and (**f**): July) analysis plots for different time periods and locations.

In this study, principal component analysis (PCA) was used to explore the response relationships between microbial class level abundances and sediment physicochemical parameters [34,35] (Figure 5a–c). The PCA results for the microbial community in October showed that the first principal component (PC1) and the second principal component (PC2) explained 53.2% and 21.1% of the total community variation, respectively. The ordination plot revealed a clear spatial separation between SS and SH along the PC1 axis. Data analysis indicated that D50, TP and TN were the core environmental factors driving community differentiation in October. In the river, the sample distribution showed a strong positive correlation with the vector directions of D50, TP, and TN. Correspondingly, in this area, *Anaerolineae* (under the phylum *Chloroflexota*) exhibited a significant abundance advantage (e.g., at point H5). In contrast, SS was distributed in the opposite direction of the above-mentioned physicochemical factor vectors, and its community composition was dominated by *Alphaproteobacteria*.

Through a comparative analysis of the data from March and July, the sediment microbial community exhibited significant seasonal succession characteristics. The PCA ordination plots showed that the samples from the two periods shifted notably along the PC2 axis, and the driving intensity of environmental factors changed with the seasons. In March (spring), data showed that the pH value across the study area was relatively high (7.08–7.41), and this relatively alkaline microenvironment maintained the stability of *Alphaproteobacteria*. At this time, the influence of D50 and OM on the community decreased compared to October, reflecting the low activity of biochemical processes under low temperature conditions. In July (summer), data showed that the relative abundance of *Anaerolineae* in river sediments in July increased by an average of 12.4% compared to March. Meanwhile, the distribution of *Gammaproteobacteria* showed a strong correlation with the fluctuating pH vector in summer.

The RDA results show that the driving mechanisms of sediment physicochemical factors on microbial community succession in March, July, and October exhibit significant seasonal differentiation (Figure 5d–f) [36]. In March (spring), the cumulative explanatory power of RDA was 23.05%, with the core driving factor being the median particle size (D50, r^2^ = 0.541, *p* = 0.009) (Table S6). In July (summer), the cumulative explanatory power increased to 39.00%, and the core driving factor shifted to organic matter (OM, r^2^ = 0.573, *p* = 0.005), with TN and TP also being significant (Table S7). In October (autumn), the RDA model achieved the highest cumulative explanatory power of 42.22% across all seasons. pH exhibited the highest explanatory contribution (r^2^ = 0.359, *p* = 0.06), followed by TP (r^2^ = 0.243, *p* = 0.176) and TN (r^2^ = 0.242, *p* = 0.174) (Table S8).

PERMANOVA based on Bray–Curtis dissimilarity was performed to quantify the effects of groups (wetland vs. river) and season on the microbial community structure (Tables S9 and S10). Global permutation tests confirmed significant community differentiation across groups [37]. SS was identified as the primary driver, explaining 14.9% of the total variation (R^2^ = 0.149, *p*-adjust = 0.014). Seasonal effects were weaker: October showed marginally significant community divergence (*p* = 0.022, *p*-adjust = 0.066), while March and July had no significant impacts (*p* > 0.05).

### 3.6. Functional Prediction of Sediment Microbial Communities

**Figure 6:**
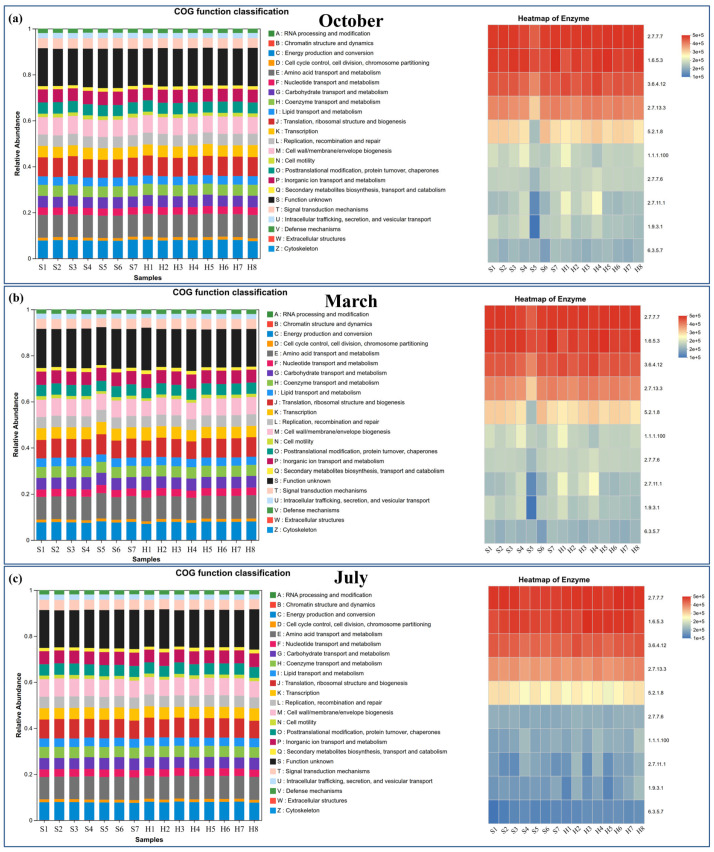
Functional prediction and gene abundance differences of sediment microbial communities at different sites across different periods: (**a**) October, (**b**) March, and (**c**) July.

In this study, PICRUSt2 was used to predict the functional profiles of sediment microbial communities across the three months. Notably, such results only reflect putative functional potential rather than in situ microbial metabolic activity, without representing actual gene expression or real-time physiological status [38] (Figure 6). Systematic inter-group comparative analysis was strictly performed between SH and SS groups to quantitatively clarify habitat-driven and seasonal divergent patterns of microbial functional traits. At the KEGG Level 1 functional level, metabolism was the dominant microbial function across the study area in all three months (greater than 70%). In October, among the COG functional categories, the basic biological functions of [E] Amino acid transport and metabolism (average abundance 8.92%), [L] Replication, recombination and repair (7.84%), and [C] Energy production and conversion (6.45%) were predominant. Further KEGG Level 2 inter-group comparative analysis verified significant habitat-specific divergence in microbial functional potential between the SH and SS groups. Specifically, the SH exhibited higher predicted functional gene abundances associated with carbohydrate metabolism and amino acid metabolism than the wetland group. Meanwhile, [G] Carbohydrate transport and metabolism (6.12%) in the COG classification, which is associated with heterotrophic metabolism, showed clear enrichment in the river area. SS had higher predicted abundances in the categories of membrane transport and signal transduction. In addition, [T] Signal transduction mechanisms (5.28%) in the COG classification also showed a slight advantage.

In March, among the COG functional categories, the top three in abundance were [E] Amino acid transport and metabolism (8.97%), [L] Replication, recombination and repair (7.81%), and [C] Energy production and conversion (6.41%). Comparative analysis at the Level 2 metabolic pathway and enzyme level showed functional differentiation among habitats. SH exhibited higher predicted gene abundances in carbohydrate metabolism and xenobiotic biodegradation and metabolism pathways. At the enzyme level, hydrolases related to carbon source utilization were significantly enriched in the river area. SS maintained a higher predicted potential for membrane transport and signal transduction. Meanwhile, [T] Signal transduction mechanisms (5.32%) in the COG classification showed a slight advantage in the wetland.

In July, the COG functional categories with the highest abundances were [E] Amino acid transport and metabolism (9.15%), [L] Replication, recombination and repair (7.92%), and [C] Energy production and conversion (6.58%). Comparative analysis at the Level 2 metabolic pathway and enzyme level revealed significant habitat specificity. Under the high temperature environment in July, SH showed the highest annual predicted gene abundances related to carbohydrate metabolism, amino acid metabolism, and energy metabolism. Enzyme-level analysis showed that hydrolases and transferases, which are closely related to organic matter degradation, were significantly enriched in the river area. SS maintained a higher predicted potential for membrane transport and signal transduction. Meanwhile, [T] Signal transduction mechanisms (5.41%) in the COG classification exhibited higher adaptive potential in the wetland cluster.

## 4. Discussion

### 4.1. N Adsorption Characteristics and Controlling Factors

The sediment adsorption capacity showed significant vertical and seasonal differences, with surface > middle > bottom, and March > July > October. This is mainly because of the low water temperature in spring, which is thermodynamically more favorable for the exothermic sorption reaction [39]. Meanwhile, microbial degradation activity was relatively low in early spring, leaving a large number of vacant adsorption sites on the sediment surface [40]. Summer warming reduced physical adsorption but enhanced biological processes mediated by plant roots and microbes. In autumn, adsorption sites approached saturation, leading to the lowest capacity.

### 4.2. Spatial and Seasonal Variations in N Source–Sink Patterns

SS acted as a stable N sink, while SHs were persistent N sources, particularly in July and October. Low temperatures in March reduced microbial mineralization and N accumulation, weakening the source effect of rivers. High temperatures promoted the ammonification of sediment organic N, enhancing the diffusion potential from the solid phase to the liquid phase [41]. After summer accumulation, river sediments maintained high diffusion flux in autumn [42]. The formation of this source and sink pattern can be attributed to the following main reasons: (1) The root action of wetland plants and the finer sediment particles (D50, Table S2) resulted in very low ENC_0_ values in SS. (2) Long-term external inputs to the river areas led to near-saturation of sediment sorption sites, thereby raising ENC_0_. Under changing environmental conditions, these sites quickly transformed into N sources and became major contributors to the nitrogen load in the water body.

In addition, due to the low water temperature and limited biological activity, there was clear competition for sorption sites in some heavily polluted river areas (e.g., H5) in spring (March). When a large amount of NH_3_-N was released and occupied the active sites on the sediment surface, it further weakened the sediment’s ability to retain other elements, such as phosphorus, leading to a simultaneous sharp increase in the release intensity of N.

### 4.3. Succession of Microbial Diversity and Community Composition

Microbial diversity was significantly higher in warm seasons (July and October) than in spring, consistent with higher organic matter mineralization and nutrient availability. This is attributed to the complex plant root environment in the wetland, which provides diverse microhabitats and a stable supply of organic carbon, thereby enhancing the resilience of the microbial community [43,44]. Particularly in the water inlet area, severe disturbance from fluctuations in the external water quality impaired the evenness of the microbial community, resulting in a generally lower Shannon index compared to the wetland interior.

*Proteobacteria*, *Bacteroidota*, *Acidobacteriota*, and *Chloroflexota* dominated across seasons. *Gammaproteobacteria* was generally highly associated with the cycling of nitrogen [45]. At the river inlet and surrounding areas (e.g., H3 and S7), due to higher nutrient loads, the dominance of this class was even more pronounced. *Anaerolineae* was highly enriched in river sediments, especially in summer and autumn. This distribution pattern reflects the adaptation of microorganisms to spring environmental conditions. Because the temperature rise in March was relatively slow, the decomposition rate of the accumulated organic matter in the sediment was limited, allowing *Anaerolineae*, which participate in the degradation of complex organic compounds, to dominate at some sites. Meanwhile, the detection of *Cyanobacteriia* at sites such as H4 may indicate that photosynthetic microorganisms in the surface water began to input organic matter into the sediment interface during spring, thereby influencing microbial succession in the surface sediment. The increase in *Cyanobacteriia* reflects the high primary productivity of the wetland system in summer.

### 4.4. Seasonal Environmental Driving Mechanisms

PCA and RDA confirmed significant seasonal shifts in driving factors. The sediment physical structure (D50) dominated in spring. OM, TN and TP became dominant in summer. pH and particle structure co-drove community variation in autumn, with increasing explanatory power across seasons.

In spring (March), D50 emerged as the primary driver of community variation, while nutrient factors (TN and TP) played a secondary role. Microbial communities were relatively concentrated in the ordination space, indicating low structural heterogeneity. Notably, river sediment samples showed a strong positive correlation with D50, TN, and TP, whereas wetland samples were distributed in the opposite direction, reflecting that sediment physical properties rather than nutrient levels governed community assembly during this period.

In summer (July), OM, TN, and TP became the dominant drivers, with their vector lengths increasing significantly alongside rising water temperatures. SH shifted strongly toward the positive PC1 axis, correlating with intensified endogenous metabolic activity under high-temperature conditions. In contrast, wetland samples showed a marked negative correlation with pH, indicating that elevated organic loading and nutrient enrichment drove microbial communities toward an eutrophic succession trajectory. The enhanced community heterogeneity in river habitats further suggests that high summer temperatures amplify the filtering effect of sediment physicochemical conditions on anaerobic microbial assemblages.

In autumn (October), habitat heterogeneity, mediated by the combined effects of sediment pH and particle structure, exerted the strongest influence on microbial spatial differentiation. Ordination revealed clear habitat partitioning: pH and D50 oriented toward the wetland group, while TP, TN, and OM aligned with the river group. The fine particle characteristics and high nutrient accumulation in river sediments provided specific ecological niches for anaerobic metabolic flora, leading to a community structure that deviated significantly from the stable, oligotrophic state observed in wetlands. Across seasons, the cumulative explanatory power of environmental factors increased progressively, reflecting the growing complexity of community–environment interactions from spring to autumn.

### 4.5. Microbial Functional Differentiation Between Groups

Metabolic functions dominated the microbial functional profiles in all sediments, reflecting the essential role of microbial metabolism in nutrient cycling at the sediment–water interface. Significant spatial differentiation in the predicted functional potential was observed between the wetland and river habitats. SHs were characterized by enhanced heterotrophic metabolism, carbohydrate metabolism, and enzyme activities related to organic matter decomposition, especially in July. These functions correspond to a high organic load, rapid organic matter mineralization, and strong nitrogen release risk in river sections, consistent with the observed N source behavior. The high abundance of hydrolases and transferases further supports intensive organic matter degradation and nutrient regeneration in SH. In contrast, SS maintained stable and high functional potential in membrane transport and signal transduction throughout the year. This functional strategy helps wetlands sense environmental changes, regulate nutrient uptake and retention, and maintain system homeostasis. Such functional characteristics underpin the steady N sink role of wetlands and enhance their buffering capacity against external nutrient inputs.

Seasonally, the microbial functional potential in rivers peaked in July, corresponding to a high temperature, vigorous microbial activity, and the strongest internal N release. The functional structure in spring and autumn was relatively stable, reflecting lower metabolic intensity under lower temperatures [46]. In addition, Mantel tests further quantified the significant associations between specific functional categories and nitrogen dynamics (Figure S4) [47]: In March, pH was negatively correlated with OM and positively correlated with ENC_0_. By comparison, in October, ENC_0_ was positively correlated with OM, TN and TP, implying enhanced N release potential under high nutrient loading. Mantel tests indicated that microbial pathways, including energy metabolism, amino acid metabolism, and inorganic ion transport, were significantly negatively correlated with TN in March. This demonstrates that the vigorous microbial metabolic and ion transport functions in early spring effectively suppressed endogenous N release, strengthened sediment N retention, and stabilized the N sink function of the sediments. Collectively, environmental filtering shapes microbial functional succession, and differentiated functional traits in turn mediate N source–sink transitions: heterotrophic and catabolic functions in rivers promote N release under warm, high organic matter conditions, while stable homeostatic functions in wetlands sustain N retention. The seasonal succession of microbial functional profiles was thus temporally and functionally coupled with the dynamics of sediment N source–sink conversion, forming a coherent abiotic–biotic regulatory loop of endogenous N cycling.

## 5. Conclusions

N adsorption capacity and source–sink functions exhibited spatial and seasonal heterogeneity between wetland sediments (SS) and river sediments (SH). SS consistently acts as a stable N sink, exhibiting high NH_3_-N sorption capacity and retention intensity, while some SH sites play the role of a N source in autumn (October). The dominant phyla were Proteobacteria, Bacteroidota, and Acidobacteriota. The microbial community functions of SS inferentially maintain steady-state regulation centered on signal transduction and membrane transport, thereby effectively buffering external nitrogen loads. In contrast, the microbial community functions of SH evolve from reserve-type heterotrophic metabolism to high-activity energy metabolism. D50 dominated in spring, OM and nutrient variables (TN and TP) jointly shaped community variation in summer, and pH interacted synergistically with the sediment physical structure to regulate spatial differentiation in autumn. The cumulative explanatory power of RDA models increased from 23.05% (March) to 39.00% (July) and 42.22% (October). These results indicate that SH is an active functional zone with a high organic load and N release risk, while SS serves as a stabilization and buffering zone, inferring the coupling mechanism whereby the seasonal succession of sediment physicochemical properties and habitat heterogeneity jointly drive the spatial differentiation of microbial community structure and function. This study lays a foundation for future research, including long-term monitoring of nutrient fluxes at the wetland–river interface and sediment N source–sink conversion, and the formulation of targeted strategies to reduce endogenous nutrient release.

## Data Availability

The original contributions presented in this study are included in the article/Supplementary Material. Further inquiries can be directed to the corresponding authors.

## Supplementary Materials

The following supporting information can be downloaded at: https://www.mdpi.com/article/10.3390/microorganisms14061216/s1, Figure S1. Sample Site location map. Figure S2. Differences in sampling point Q_max_ and ENC_0_ across different periods: Q_max_ of wetland in October (a), March (b), July (c); Q_max_ of river in October (d), March (e), July (f). Figure S3. Cladograms showing the differences in relative abundance of bacterial communities in the three seasons according to the LEfSe analysis (LDA > 3, *p* < 0.05). Differences are represented by the color (March: spring, July: summer, October: autumn). The diameter of each circle is proportional to a taxon’s abundance. The circles from the inner region to the outer region represent the phylogenetic levels from domain to genus. Figure S4. Mantel test of seasonal linkages between environmental variables and microbial functional potential. Table S1. Coordinates of Sample Site. Table S2. Physicochemical properties of overlying water and sediments. Table S3. NH_3_-N sorption parameters using modified Langmuir and Freundlich models in October 2024. Table S4. NH_3_-N sorption parameters using modified Langmuir and Freundlich models in March 2025. Table S5. NH_3_-N sorption parameters using modified Langmuir and Freundlich models in July 2025. Table S6. The RDA parameters (March). Table S7. The RDA parameters (July). Table S8. The RDA parameters (October). Table S9. PERMANOVA results grouped by sample site (SS/SH). Table S10. PERMANOVA results grouped by season (October/March/July). References [48,49,50,51,52,53,54,55,56,57,58] are cited in the Supplementary Materials.
