# Supplementary material for: Temporal–Spatial Differences of Nitrogen Source–Sink in Sediments of Wetland–River Connected System and Response Mechanism of Microbial Community Function
Source: Microorganisms. 2026 May 27;14(6):1216. doi: 10.3390/microorganisms14061216 (PMC13302948; doi:10.3390/microorganisms14061216)
Supplement: Supplementary file 1 [file microorganisms-14-01216-s001.zip › microorganisms-4284102-supplementary.pdf]

Supplementary Materials

Zejun Shi [a], Yu Pan [a], Haojie Chen [a, b], Xueying Wang [b], Wei Huang [b*], Lixin Li [a*]

[a] *School of Environment and Chemical Engineering, Heilongjiang University of Science and Technology, Harbin, 150022, China*

[b] *State Environmental Protection Engineering Center for Pollution Treatment and Control in Textile Industry, College of Environmental Science and Engineering, Donghua University, Shanghai, 201620, PR China;*

**Corresponding author: Wei Huang @ Lixin Li*

E-mail address: huangwei@dhu.edu.cn (H.Wei)

lilixin1980@163.com (L. Li)

S1 Sample Analysis Method

Fresh sediment samples were centrifuged to obtain pore water samples, while the remaining sediments were first dried using a freeze dryer, ground, and sieved through a 100-mesh sieve. Phosphorus (P) forms in sediments were fractionated using the SMT (Standards, Measurements and Testing) method[S1]. The median particle size (D50) of sediments was determined using a laser particle size analyzer. Particle size distribution characteristics directly influence the specific surface area of sediments and their pollutant adsorption capacity[S2]. Total nitrogen (TN) in sediments was measured by alkaline potassium persulfate digestion-ultraviolet spectrophotometry[S3]. Inorganic nitrogen in sediments was determined by KCl solution extraction-spectrophotometry[S4]. Exchangeable nitrogen in sediments was extracted, with separate measurements of ammonium nitrogen (NH_4^+-N) and nitrate nitrogen (NO_3^--N) to reflect nitrogen transformation processes and activity levels. Organic matter (OM) content in sediments was determined by the burn-off loss method (550 °C, 4 h)[S5],

S2 N sorption isotherm experiment

This study conducted adsorption isothermal experiments for nitrogen (N). Sediment samples (1 g) from each sampling site, after drying and grinding, were quantitatively transferred into 50 mL centrifuge tubes. Subsequently, 40 mL NH_4Cl solution (concentration series: 0, 1, 2, 5, 10, 20, 50 mg/L) was added according to the experimental design gradient. The samples were placed in a constant temperature shaker with a temperature set at (25±2) °C and oscillated at 180 rpm for 18 hours. After centrifugation and filtration through a 0.45 μm polyether sulfone filter, the NH_3-N concentration in the supernatant was analyzed[S6].

S3 Data analysis

The adsorption isotherms of NH3-N by sediments are typically described using the Langmuir and Freundlich models. The modified Langmuir isothermal adsorption model is given as follows:[S7-S9]

$$Q_e=\frac{Q_{max}K_LC_e}{1+K_LC_e}-\frac{Q_{max}K_LC_e^0}{1+K_LC_e^0}-Q_e^0$$

The modified Freundlich isothermal sorption model is:

$$Q_e=K_FC_e^n-K_F(C_e^0)^n-Q_e^0$$

$$NAN=\frac{Q_{max}K_LC_e^0}{1+K_LC_e^0}+Q_e^0$$

$$ENC_0=\frac{NAP}{K_L(Q_{max}-NAN)}$$

Q_{max} denotes the theoretical maximum adsorption capacity of the modified Langmuir model, expressed in mg/g; K_L represents the adsorption coefficient, measured in L/mg; C_e^0 indicates the solution NH_3-N concentration at adsorption equilibrium after adding deionized water, measured in mg/L; Q_e^0 refers to the NH_3-N adsorption capacity of the sediment at adsorption equilibrium after adding deionized water, measured in mg/g; K_F is the adsorption coefficient, expressed in L/g; n is a constant.

ENC_0 is the nitrogen level (mg/L) in the solution at the point of dynamic

equilibrium within the adsorbent system. NAN (mg/g) refers to the combined quantity of exchangeable nitrogen in the equilibrated solution and the ammonia nitrogen attached to the substrate surface

The N source and sink state determination by the difference between ENC_0 with NH_3-N concentration in overlying water, and the equation was as follows:

$$\delta = ENC_0 - CNH_3\text{-}N$$

CNH_3-N was the NH_3-N concentration in overlying water, and δ was the difference between ENC_0 and CNH_3-N. When $\delta < 0$, the sediment is a “source” of N, and when $\delta > 0$, the sediment is a “sink” of N.

The richness estimators (Chao) and the diversity indices (Shannon) were calculated. Principal component analysis (PCA) was employed to explore and visualize the similarities between sediment samples obtained from four seasons based on Bray-Curtis dissimilarity using the package Ape [S10]. Canoco 5 software was used to carry out redundancy analysis (RDA) based on population abundance and environmental factors. The sediment samples with specific indicator groups of bacteria were found using LEfSe (Linear discriminant analysis Effect Size) [S11]. Independent T-test and permutational multivariate analysis of variance were used to determine the differences that exist among different groups.

5 Figures

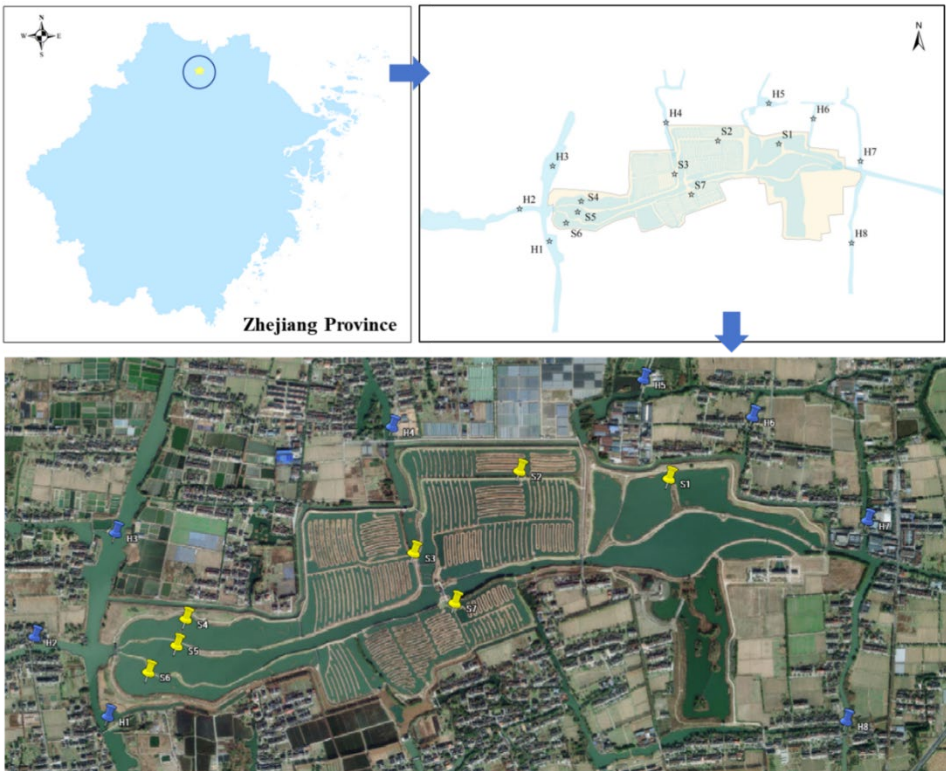

Figure S1. Sample Site location map.

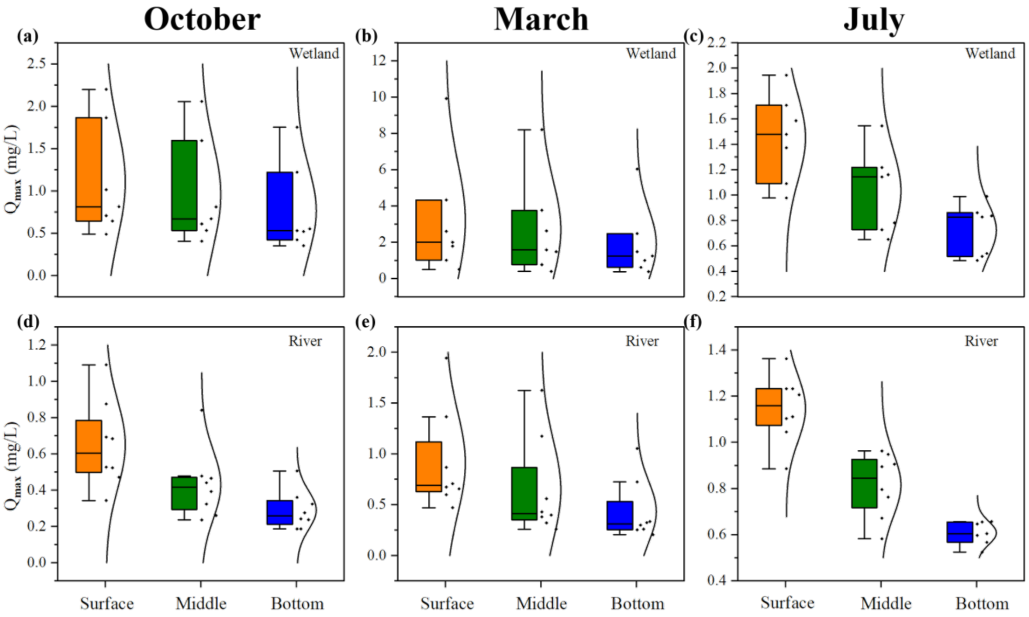

Figure S2. Differences in sampling point Q_{max} and ENC_0 across different periods: Q_{max} of wetland in October (a), March (b), July (c); Q_{max} of river in October (d), March (e), July (f).

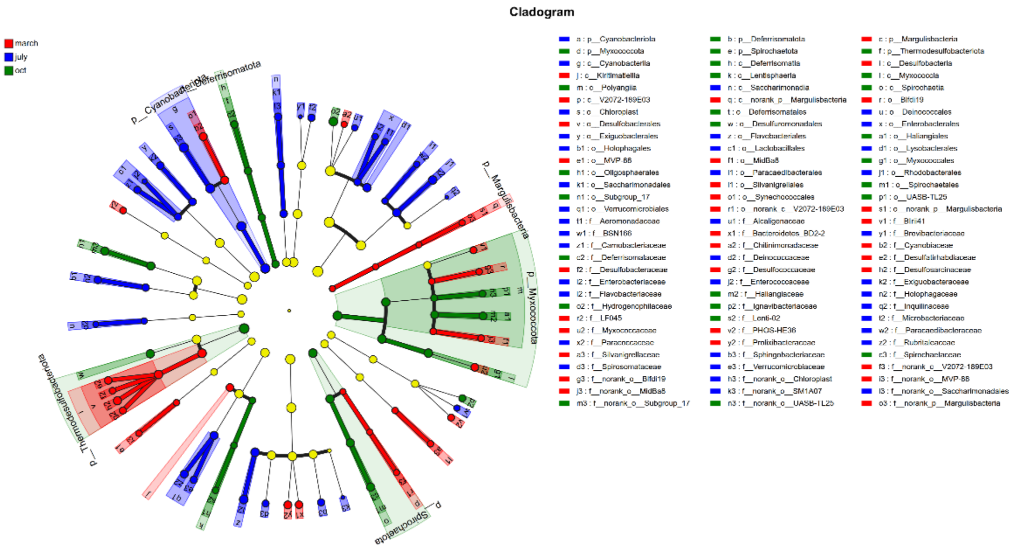

Figure S3. Cladograms showing the differences in relative abundance of bacterial communities in the three seasons according to the LEfSe analysis (LDA > 3, $p < 0.05$). Differences are represented by the color (March: spring, July: summer, October: autumn). The diameter of each circle is proportional to a taxon's abundance. The circles from the inner region to the outer region represent the phylogenetic levels from domain to genus.

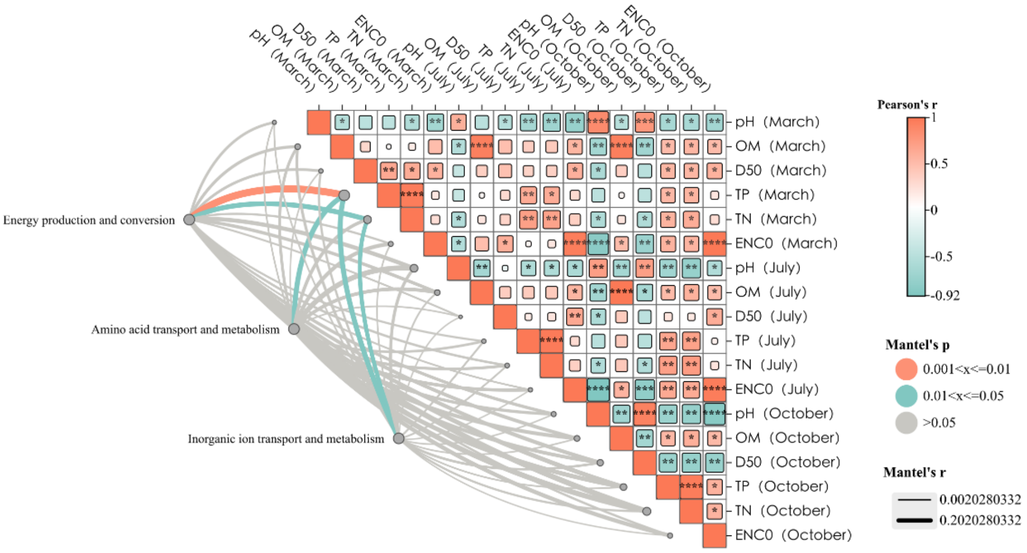

Figure S4. Mantel test of seasonal linkages between environmental variables and microbial functional potential.

6 Tables

Table S1. Coordinates of Sample Site.

Sample Site	longitude	latitude
**S1**	120.3381872	30.54077016
**S2**	120.3297973	30.54113977
**S3**	120.3238267	30.53718482
**S4**	120.3109896	30.53396898
**S5**	120.3105068	30.53269371
**S6**	120.3089082	30.5313907
**S7**	120.3261495	30.53476371
**H1**	120.306623	30.52921897
**H2**	120.3025031	30.53306336
**H3**	120.3070521	30.53814585
**H4**	120.3226519	30.54330199
**H5**	120.3367925	30.54557505
**H6**	120.3429186	30.543764
**H7**	120.3494203	30.53872339
**H8**	120.3481972	30.52901566

Table S2. Physicochemical properties of overlying water and sediments.

Sampling Sites	Date	pH (Overlying water)	pH (Sediment)	DO (mg/L) (Overlying water)	D50 (Sediment)
S1	Oct-24	7.72	7.05	8.04	26.53
	Mar-25	7.88	7.21	9.51	13.58
	Jul-25	8.22	7.49	7.82	18.12
S2	Oct-24	7.62	7.02	8.17	21.12
	Mar-25	7.78	7.23	7.73	19.83
	Jul-25	8.12	7.17	5.69	13.56
S3	Oct-24	7.55	7.01	7.90	19.64
	Mar-25	7.71	7.41	7.34	13.91
	Jul-25	8.05	7.19	5.92	12.81
S4	Oct-24	7.36	6.92	6.11	16.71

	Mar-25	7.52	7.26	7.81	12.36
	Jul-25	7.86	7.26	6.48	10.89
S5	Oct-24	7.38	6.93	6.49	17.19
	Mar-25	7.54	7.08	7.75	11.16
	Jul-25	7.88	7.13	6.32	10.36
S6	Oct-24	7.43	6.86	6.24	15.94
	Mar-25	7.59	7.17	7.82	14.86
	Jul-25	7.93	7.03	6.17	12.64
S7	Oct-24	7.57	6.97	7.62	17.29
	Mar-25	7.73	7.27	7.44	15.49
	Jul-25	8.07	7.43	6.27	12.69
H1	Oct-24	7.31	6.68	6.11	12.41
	Mar-25	7.47	6.82	6.3	17.16
	Jul-25	7.81	6.85	5.08	9.05
H2	Oct-24	7.25	6.66	6.54	11.57
	Mar-25	7.41	6.97	6.11	13.82
	Jul-25	7.75	6.94	4.86	21.93
H3	Oct-24	7.26	6.65	6.25	10.1
	Mar-25	7.42	6.78	5.44	19.61
	Jul-25	7.76	7.19	3.71	28.89
H4	Oct-24	7.34	6.74	6.08	12.81
	Mar-25	7.51	6.99	6.75	12.23
	Jul-25	7.84	7.08	5.31	17.19
H5	Oct-24	7.12	6.52	6.87	9.05
	Mar-25	7.28	6.87	5.05	23.17
	Jul-25	7.62	7.01	3.16	32.81
H6	Oct-24	7.15	6.55	7.35	9.64
	Mar-25	7.31	6.94	5.67	25.47
	Jul-25	7.65	6.9	3.66	16.71
H7	Oct-24	7.51	6.81	6.24	14.42
	Mar-25	7.67	7.06	7.08	19.92
	Jul-25	8.01	7.03	5.74	21.32
H8	Oct-24	7.46	6.76	6.38	13.36
	Mar-25	7.62	6.95	7.21	19.72
	Jul-25	7.96	7.13	5.52	14.81

Table S3. NH_3-N sorption parameters using modified Langmuir and Freundlich models in October 2024.

Layers	Sites	Modified Langmuir model			Modified Freundlich model		
		R^2	Q_{max}/(mg·g^{-1})	K_L/(L·mg^{-1})	R^2	K_F/(L·g^{-1})	n
Surface	S1	0.995	2.201±0.131	0.052±0.007	0.965	0.219±0.061	0.521±0.076
	S2	0.996	1.865±0.086	0.065±0.007	0.966	0.238±0.062	0.475±0.071
	S3	0.986	0.707±0.049	0.105±0.021	0.935	0.145±0.049	0.387±0.088
	S4	0.983	0.645±0.042	0.149±0.031	0.918	0.183±0.063	0.329±0.086
	S5	0.988	0.814±0.065	0.063±0.012	0.958	0.102±0.031	0.477±0.081
	S6	0.991	0.489±0.021	0.198±0.029	0.938	0.173±0.047	0.292±0.064
	S7	0.995	1.014±0.061	0.045±0.005	0.976	0.089±0.021	0.541±0.065
	H1	0.979	0.692±0.083	0.053±0.014	0.941	0.069±0.026	0.522±0.104
	H2	0.959	0.343±0.065	0.048±0.021	0.925	0.031±0.014	0.546±0.128
	H3	0.972	0.875±0.126	0.054±0.018	0.942	0.093±0.034	0.513±0.102
	H4	0.945	0.527±0.096	0.072±0.035	0.925	0.083±0.034	0.439±0.111
	H5	0.969	0.684±0.131	0.036±0.014	0.979	0.061±0.013	0.531±0.062
	H6	0.940	0.524±0.133	0.043±0.024	0.955	0.054±0.018	0.507±0.091
	H7	0.970	1.091±0.311	0.021±0.011	0.980	0.046±0.012	0.649±0.071
	H8	0.931	0.472±0.123	0.047±0.028	0.944	0.055±0.021	0.487±0.101
Middle	S1	0.996	2.058±0.123	0.036±0.004	0.982	0.141±0.031	0.584±0.059
	S2	0.995	1.594±0.087	0.055±0.007	0.980	0.173±0.035	0.503±0.055
	S3	0.986	0.611±0.043	0.103±0.021	0.932	0.122±0.042	0.393±0.091
	S4	0.988	0.533±0.031	0.135±0.023	0.927	0.138±0.046	0.344±0.084
	S5	0.985	0.671±0.057	0.071±0.015	0.942	0.094±0.032	0.458±0.093

	S6	0.992	0.407±0.015	0.225±0.031	0.894	0.147±0.051	0.292±0.081
	S7	0.992	0.811±0.061	0.053±0.009	0.961	0.082±0.024	0.517±0.082
	H1	0.986	0.478±0.044	0.056±0.012	0.959	0.052±0.016	0.504±0.084
	H2	0.954	0.236±0.051	0.042±0.019	0.922	0.017±0.008	0.577±0.137
	H3	0.965	0.841±0.208	0.028±0.013	0.965	0.048±0.015	0.613±0.089
	H4	0.912	0.324±0.073	0.083±0.053	0.921	0.068±0.027	0.387±0.106
	H5	0.954	0.441±0.093	0.045±0.021	0.977	0.055±0.012	0.467±0.061
	H6	0.911	0.393±0.137	0.038±0.028	0.943	0.041±0.015	0.511±0.105
	H7	0.936	0.465±0.111	0.052±0.029	0.936	0.056±0.022	0.487±0.107
	H8	0.908	0.261±0.071	0.059±0.041	0.957	0.052±0.014	0.396±0.076
Bottom	S1	0.992	1.752±0.144	0.041±0.006	0.972	0.131±0.034	0.571±0.072
	S2	0.995	1.222±0.061	0.071±0.008	0.967	0.169±0.042	0.461±0.067
	S3	0.984	0.531±0.041	0.101±0.021	0.924	0.102±0.037	0.401±0.097
	S4	0.982	0.423±0.028	0.158±0.033	0.903	0.123±0.045	0.326±0.092
	S5	0.979	0.521±0.048	0.091±0.022	0.917	0.091±0.036	0.417±0.105
	S6	0.994	0.352±0.012	0.162±0.018	0.941	0.104±0.028	0.326±0.067
	S7	0.989	0.551±0.041	0.071±0.013	0.946	0.075±0.025	0.462±0.089
	H1	0.982	0.361±0.033	0.077±0.018	0.936	0.054±0.019	0.445±0.096
	H2	0.922	0.187±0.086	0.022±0.017	0.909	0.006±0.004	0.707±0.181
	H3	0.945	0.506±0.108	0.053±0.027	0.937	0.062±0.024	0.485±0.106
	H4	0.925	0.242±0.053	0.071±0.042	0.953	0.051±0.015	0.385±0.077
	H5	0.940	0.275±0.065	0.047±0.026	0.984	0.045±0.007	0.418±0.045
	H6	0.921	0.237±0.057	0.063±0.039	0.952	0.047±0.014	0.394±0.078
	H7	0.953	0.323±0.061	0.058±0.027	0.961	0.052±0.014	0.431±0.073

	H8	0.916	0.187±0.042	0.081±0.049	0.943	0.047±0.014	0.361±0.079

Table S4. NH_3-N sorption parameters using modified Langmuir and Freundlich models in March 2025.

Layers	Sites	Modified Langmuir model			Modified Freundlich model		
		R^2	Q_{max}/(mg·g^{-1})	K_L/(L·mg^{-1})	R^2	K_F/(L·g^{-1})	n
Surface	S1	0.999	9.931±1.016	0.006±0.001	0.999	0.084±0.004	0.855±0.013
	S2	0.997	4.331±0.400	0.017±0.002	0.992	0.121±0.024	0.724±0.054
	S3	0.997	2.599±0.483	0.009±0.002	0.994	0.031±0.005	0.835±0.047
	S4	0.995	1.013±0.042	0.089±0.009	0.954	0.181±0.053	0.416±0.076
	S5	0.999	1.795±0.103	0.016±0.001	0.998	0.047±0.004	0.726±0.022
	S6	0.992	0.502±0.019	0.203±0.031	0.927	0.170±0.053	0.325±0.068
	S7	0.999	2.002±0.138	0.015±0.002	0.994	0.045±0.007	0.755±0.042
	H1	0.955	1.943±1.639	0.008±0.001	0.951	0.021±0.014	0.847±0.178
	H2	0.994	0.673±0.133	0.012±0.003	0.989	0.011±0.003	0.800±0.065
	H3	0.992	1.365±0.171	0.024±0.005	0.979	0.058±0.017	0.662±0.077
	H4	0.988	0.598±0.043	0.077±0.014	0.944	0.092±0.031	0.440±0.088
	H5	0.997	0.707±0.041	0.038±0.005	0.989	0.058±0.011	0.547±0.046
	H6	0.988	0.655±0.079	0.033±0.008	0.993	0.053±0.001	0.541±0.003
	H7	0.998	0.868±0.047	0.031±0.003	0.992	0.053±0.008	0.597±0.044
	H8	0.996	0.472±0.024	0.057±0.007	0.971	0.056±0.014	0.488±0.069
Middle	S1	0.999	8.210±0.626	0.006±0.001	0.999	0.067±0.003	0.862±0.013

	S2	0.996	3.767±0.483	0.013±0.002	0.993	0.070±0.013	0.779±0.049
	S3	0.997	2.627±0.658	0.007±0.002	0.995	0.022±0.004	0.871±0.045
	S4	0.981	0.762±0.062	0.105±0.024	0.921	0.157±0.058	0.389±0.096
	S5	0.997	1.584±0.185	0.013±0.002	0.994	0.030±0.005	0.773±0.045
	S6	0.996	0.393±0.011	0.194±0.019	0.925	0.130±0.038	0.308±0.071
	S7	0.992	1.477±0.217	0.019±0.005	0.981	0.043±0.011	0.726±0.074
	H1	0.959	1.627±1.448	0.007±0.008	0.954	0.014±0.009	0.879±0.166
	H2	0.985	0.380±0.107	0.013±0.006	0.978	0.007±0.002	0.786±0.092
	H3	0.995	1.175±0.135	0.019±0.004	0.992	0.043±0.008	0.674±0.050
	H4	0.979	0.320±0.027	0.117±0.029	0.934	0.082±0.029	0.353±0.085
	H5	0.991	0.428±0.033	0.055±0.011	0.985	0.056±0.012	0.461±0.053
	H6	0.958	0.399±0.087	0.039±0.018	0.954	0.042±0.018	0.486±0.108
	H7	0.985	0.560±0.066	0.043±0.011	0.975	0.056±0.017	0.509±0.076
	H8	0.988	0.259±0.019	0.084±0.016	0.945	0.049±0.017	0.357±0.081
Bottom	S1	0.996	6.043±1.369	0.007±0.002	0.994	0.056±0.010	0.862±0.050
	S2	0.992	2.486±0.391	0.017±0.004	0.985	0.065±0.015	0.735±0.065
	S3	0.996	1.484±0.314	0.009±0.003	0.993	0.017±0.004	0.839±0.055
	S4	0.984	0.613±0.044	0.109±0.022	0.937	0.134±0.046	0.381±0.085
	S5	0.994	0.998±0.122	0.019±0.004	0.988	0.031±0.006	0.709±0.056
	S6	0.998	0.379±0.009	0.113±0.008	0.975	0.085±0.016	0.373±0.050
	S7	0.994	1.243±0.171	0.017±0.003	0.984	0.031±0.008	0.749±0.068
	H1	0.982	1.052±0.547	0.008±0.005	0.978	0.011±0.004	0.865±0.104
	H2	0.961	0.252±0.124	0.013±0.009	0.953	0.005±0.002	0.789±0.143
	H3	0.973	0.725±0.153	0.029±0.012	0.969	0.049±0.019	0.568±0.099

H4	0.981	0.299±0.031	0.069±0.018	0.969	0.052±0.015	0.412±0.071
H5	0.961	0.259±0.041	0.063±0.024	0.976	0.052±0.013	0.374±0.058
H6	0.916	0.323±0.112	0.037±0.027	0.924	0.044±0.024	0.419±0.131
H7	0.969	0.334±0.048	0.061±0.021	0.957	0.055±0.019	0.419±0.088
H8	0.973	0.204±0.021	0.101±0.029	0.945	0.049±0.017	0.357±0.081

Table S5. NH_3-N sorption parameters using modified Langmuir and Freundlich models in July2025.

Layers	Sites	Modified Langmuir model			Modified Freundlich model		
		R^2	Q_{max}/(mg·g^{-1})	K_L/(L·mg^{-1})	R^2	K_F/(L·g^{-1})	n
Surface	S1	0.965	1.708±0.441	0.026±0.012	0.953	0.075±0.031	0.667±0.114
	S2	0.981	1.091±0.158	0.037±0.011	0.972	0.082±0.022	0.569±0.074
	S3	0.985	1.372±0.209	0.029±0.008	0.971	0.074±0.022	0.626±0.081
	S4	0.995	0.977±0.051	0.058±0.007	0.977	0.118±0.025	0.485±0.058
	S5	0.997	1.944±0.071	0.071±0.006	0.972	0.263±0.062	0.465±0.064
	S6	0.994	1.479±0.103	0.042±0.006	0.986	0.128±0.022	0.539±0.048
	S7	0.983	1.586±0.224	0.036±0.011	0.961	0.104±0.035	0.595±0.092
	H1	0.988	0.886±0.082	0.051±0.011	0.952	0.075±0.027	0.555±0.098
	H2	0.983	1.232±0.152	0.043±0.011	0.985	0.112±0.001	0.528±0.003
	H3	0.994	1.362±0.096	0.044±0.007	0.980	0.107±0.024	0.561±0.061
	H4	0.996	1.045±0.049	0.061±0.007	0.977	0.113±0.025	0.504±0.061
	H5	0.998	1.103±0.031	0.089±0.007	0.961	0.172±0.046	0.438±0.072

	H6	0.983	1.232±0.152	0.043±0.011	0.947	0.085±0.034	0.592±0.109
	H7	0.977	1.111±0.161	0.043±0.014	0.939	0.077±0.033	0.591±0.119
	H8	0.975	1.206±0.183	0.043±0.014	0.935	0.082±0.037	0.596±0.124
	S1	0.947	1.218±0.396	0.027±0.016	0.939	0.056±0.027	0.659±0.131
	S2	0.968	0.728±0.117	0.051±0.018	0.953	0.077±0.025	0.511±0.091
	S3	0.979	1.144±0.263	0.021±0.008	0.973	0.046±0.014	0.668±0.082
	S4	0.991	0.651±0.042	0.074±0.013	0.960	0.104±0.028	0.436±0.072
	S5	0.992	1.546±0.121	0.044±0.007	0.992	0.143±0.019	0.524±0.036
	S6	0.982	0.782±0.071	0.077±0.019	0.948	0.131±0.041	0.423±0.083
	S7	0.984	1.161±0.167	0.033±0.009	0.971	0.075±0.021	0.595±0.078
Middle	H1	0.985	0.582±0.063	0.049±0.012	0.950	0.047±0.017	0.563±0.103
	H2	0.983	0.963±0.145	0.033±0.009	0.954	0.048±0.019	0.647±0.109
	H3	0.980	0.948±0.126	0.043±0.012	0.946	0.067±0.027	0.585±0.109
	H4	0.995	0.672±0.032	0.072±0.009	0.976	0.086±0.019	0.473±0.061
	H5	0.989	0.796±0.048	0.099±0.018	0.935	0.132±0.046	0.427±0.094
	H6	0.984	0.895±0.095	0.052±0.013	0.943	0.077±0.031	0.551±0.107
	H7	0.978	0.763±0.101	0.051±0.015	0.936	0.062±0.026	0.561±0.117
	H8	0.956	0.905±0.203	0.038±0.018	0.911	0.051±0.029	0.629±0.155
	S1	0.950	0.862±0.231	0.034±0.018	0.937	0.054±0.024	0.607±0.123
	S2	0.951	0.485±0.092	0.058±0.027	0.933	0.064±0.024	0.472±0.105
	S3	0.973	0.827±0.197	0.024±0.011	0.969	0.041±0.012	0.637±0.085
Bottom	S4	0.986	0.517±0.039	0.085±0.018	0.955	0.096±0.026	0.409±0.073
	S5	0.992	0.991±0.066	0.061±0.011	0.983	0.132±0.023	0.462±0.048
	S6	0.991	0.541±0.032	0.082±0.013	0.976	0.103±0.021	0.401±0.052

S7	0.977	0.837±0.153	0.032±0.011	0.970	0.055±0.015	0.591±0.079
H1	0.991	0.432±0.033	0.052±0.009	0.973	0.039±0.011	0.536±0.071
H2	0.976	0.646±0.093	0.043±0.013	0.940	0.046±0.019	0.583±0.114
H3	0.983	0.655±0.089	0.037±0.011	0.955	0.039±0.014	0.611±0.101
H4	0.995	0.597±0.035	0.047±0.006	0.975	0.048±0.012	0.556±0.068
H5	0.996	0.524±0.017	0.105±0.011	0.948	0.093±0.028	0.412±0.081
H6	0.987	0.605±0.055	0.058±0.013	0.953	0.058±0.021	0.531±0.094
H7	0.985	0.567±0.053	0.063±0.015	0.942	0.062±0.023	0.503±0.102
H8	0.960	0.657±0.143	0.037±0.017	0.918	0.036±0.021	0.635±0.151

Table S6. The RDA parameters (March).

	RDA1	RDA2	r^2	P_value
pH	-0.35391	0.93528	0.05563	0.699
OM	-0.07327	0.99731	0.06237	0.671
D50	0.99759	0.0694	0.54091	0.009
TP	0.99742	0.07182	0.33176	0.089
TN	0.99997	-0.0074	0.28967	0.121

Table S7. The RDA parameters (July).

	RDA1	RDA2	r^2	P_value
pH	-0.88026	-0.47449	0.29523	0.117
OM	0.9207	-0.39026	0.57265	0.005
D50	0.92285	0.38516	0.30664	0.107
TP	0.84772	0.53044	0.39151	0.038
TN	0.87561	0.48302	0.47419	0.018

Table S8. The RDA parameters (October).

	RDA1	RDA2	r^2	P_value
pH	-0.99052	0.13735	0.35872	0.06
OM	0.97929	0.20244	0.08517	0.577
D50	-0.99241	0.12297	0.2077	0.23
TP	0.49648	-0.86805	0.24262	0.176
TN	0.73272	-0.68053	0.24188	0.174

Table S9. PERMANOVA results grouped by sample site (SS/SH).

**Characteristics**	**SumsOfSqs**	**MeanSqs**	**F_Model**	**R^2**	**P_value**	**P_adjust**
SS	0.2024	0.067447	2.39035	0.148887	**0.007**	0.014
SH	0.14581	0.0486	1.644167	0.10724	0.07	0.07

Table S10. PERMANOVA results grouped by season (October/March/July).

**Characteristics**	**SumsOfSqs**	**MeanSqs**	**F_Model**	**R^2**	**P_value**	**P_adjust**
October	0.1358	0.0679	2.33021	0.09988	**0.022**	0.066

Characteristics	SumsOfSqs	MeanSqs	F_Model	R^2	P_value	P_adjust
March	0.10313	0.05157	1.72366	0.07585	0.075	0.1125
July	0.06497	0.03248	1.05378	0.04778	0.382	0.382

Reference

[S1] Qiu, Z.; Liu, Q.; Zhang, R.; Zhan, C.; Liu, S.; Zhang, J.; Liu, H.; Xiao, W., Liu, X. Distribution characteristics and pollution assessment of phosphorus forms, TOC, and TN in the sediments of Daye Lake, Central China, *Journal of Soils and Sediments* **2023**, *23*, 1023-1036.

[S2] Zhang, T.; Yan, R.; Gui, Q.; Gao, Y.; Wang, Q., Xu, S. Fine particulate matter as a key factor promoting the spread of antibiotics in river network, *Science of The Total Environment* **2024**, *935*, 173323.

[S3] Dong, X.; Chen, H.; Chang, Y.; Yang, X.; Yang, H., Huang, W. Exploration of Phosphorus Release Characteristics in Sediments from the Plains River Network: Vertical Distribution and the Response of Phosphorus and Microorganisms, *Water* **2025**, *17*, 2196.

[S4] Esala, M.J. Deep-freezing pretreatment and time of extraction of soil samples for inorganic nitrogen determination, *Communications in Soil Science and Plant Analysis* **1994**, *25*, 651-662.

[S5] Eid, E.M.; Shaltout, K.H.; Alamri, S.A.M.; Sewelam, N.A.; Galal, T.M., Brima, E.I. Prediction models for evaluating heavy metal uptake by Pisum sativum L. in soil amended with sewage sludge, *Journal of Environmental Science and Health, Part A* **2020**, *55*, 151-160.

[S6] Murphy, J., Riley, J.P. A modified single solution method for the determination of phosphate in natural waters, *Analytica Chimica Acta* **1962**, *27*, 31-36.

[S7] Sairam Sundaram, C.; Viswanathan, N., Meenakshi, S. Uptake of fluoride by nano-hydroxyapatite/chitosan, a bioinorganic composite, *Bioresource Technology* **2008**, *99*, 8226-8230.

[S8] Bian, H.; Wang, M.; Han, J.; Hu, X.; Xia, H.; Wang, L.; Fang, C.; Shen, C.; Man, Y.B.; Wong, M.H.; Shan, S., Zhang, J. MgFe-LDH@biochars for removing ammonia nitrogen and phosphorus from biogas slurry: Synthesis routes, composite performance, and adsorption mechanisms, *Chemosphere* **2023**, *324*, 138333.

[S9] Han, B.; Butterly, C.; Zhang, W.; He, J.-z., Chen, D. Adsorbent materials for ammonium and ammonia removal: A review, *Journal of Cleaner Production* **2021**, *283*, 124611.

[S10] Paradis, E.; Claude, J., Strimmer, K. APE: Analyses of Phylogenetics and Evolution in R language, *Bioinformatics* **2004**, *20*, 289-290.

[S11] Segata, N.; Izard, J.; Waldron, L.; Gevers, D.; Miropolsky, L.; Garrett, W.S., Huttenhower, C. Metagenomic biomarker discovery and explanation, *Genome Biology* **2011**, *12*, R60.
